# Improving safety-planning in patients admitted with self-harm

**DOI:** 10.1192/bjo.2021.557

**Published:** 2021-06-18

**Authors:** Vatsala Mishra, Kathryn Hughes, Alexander Sunderland, Marilia Calcia, Martin Parsons

**Affiliations:** Mental Health Liaison Team, King's College Hospital, South London and Maudsley NHS Foundation Trust

## Abstract

**Aims:**

Self-harm is a common presentation to acute hospitals, associated with increased risk of completed suicide. Safety plans are increasingly recommended to help patients recognise and prevent escalation of self-harm behaviours.

This project aimed to improve quality and documentation of safety planning for patients admitted at an acute general hospital due to self-harm, who were assessed by Liaison Psychiatry. We aimed to increase the number of patients given written safety plans on discharge by 50%.

**Method:**

The PDSA cycle model of quality improvement was used. A retrospective audit of clinical records was conducted over 3 months to establish baseline documentation of safety planning (n = 51). A template for a self-harm crisis plan, used in other areas of the Trust, was adopted, to be adapted to each patient. A leaflet for sources of crisis support and patient feedback form were developed and distributed to clinicians in the team. Data collection was repeated one month later (n = 48). The second set of interventions involved a training session for clinicians on developing safety plans in collaboration with patients, and a poster highlighting the process to be undertaken when discharging a patient admitted with self-harm.

**Result:**

Following initial interventions, 20% of patients had completed safety plans and 50% received advice, an increase of 20% and 40% respectively. The second PDSA cycle showed increase in numbers to 38% and 67% respectively.

**Conclusion:**

Creating a crisis plan with a hospital-specific leaflet for the Liaison Psychiatry team increased the number of patients discharged with safety plans in place. 86% of patients who participated in safety-planning found the process helpful and felt likely to use the plan in future crises. This is an area of ongoing quality improvement which can be implemented in other hospitals to better equip patients with skills and support to reduce self-harm/suicide attempts.

